# Supplementary material for: Titratable Pharmacological Regulation of CAR T Cells Using Zinc Finger-Based Transcription Factors
Source: Cancers (Basel). 2021 Sep 22;13(19):4741. doi: 10.3390/cancers13194741 (PMC8507528; doi:10.3390/cancers13194741)
Supplement: Supplementary file 1 [file cancers-13-04741-s001.zip › cancers-1262191-supplementary.pdf]

Supplementary Materials

Titratable Pharmacological Regulation of CAR T Cells Using Zinc Finger-Based Transcription Factors

Bettina Kotter, Fabian Engert, Winfried Krueger, Andre Roy, Wael Al Rawashdeh, Nicole Cordes, Britta Drees, Brian Webster, Niels Werchau, Dominik Lock, Sandra Dapa, Dina Schneider, Stephan Ludwig, Claudia Rossig, Mario Assenmacher, Joerg Mittelstaet and Andrew D. Kaiser

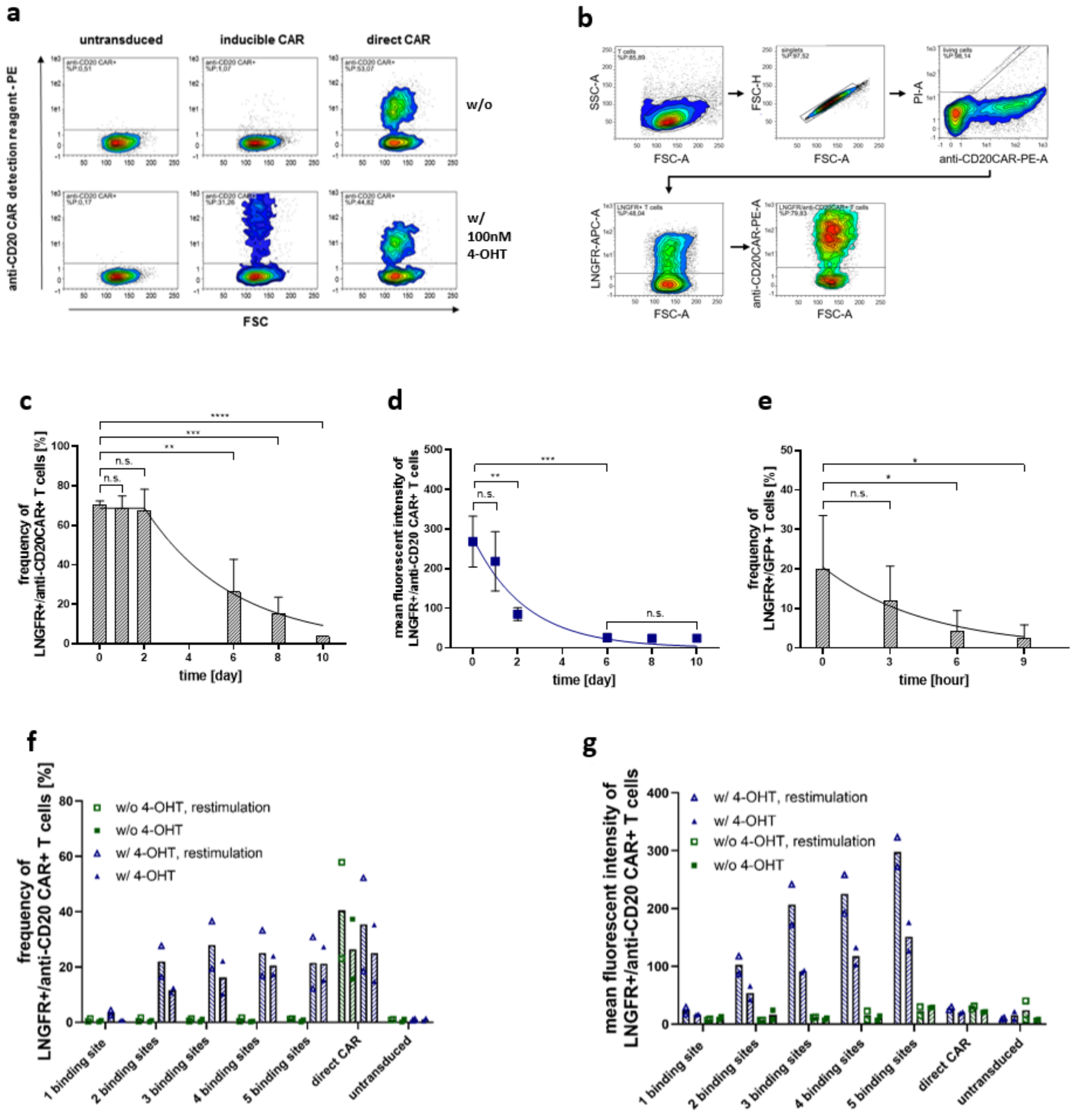

Figure S1. 4-OHT dependent induction of anti-CD20 CAR expression. (**a**) Representative flow plots show the percentage of anti-CD20 CAR positive T cells upon induction with 100 nM 4-OHT for 40 h in comparison to the conventional constitutively expressed CAR and untransduced T cells. (**b**) Cells were gated on size, singularity, viability, and ΔLNGFR expression before analyzing anti-CD20 CAR expression. (**c**) Data points from figure 1f representing the frequency of anti-CD20 CAR+ T cells following inducer drug discontinuation were fitted to a plateau followed by one-phase decay model and the half-life was calculated as t1/2≈ 2.8 days. (**d**) Data points from figure 1f representing the MFI of anti-CD20 CAR+ T cells following inducer drug discontinuation were fitted to a one-phase decay model and the half-life was calculated as t1/2≈ 1.6 days. (**e**) Data points from figure 1h representing the frequency of GFP+ T cells following inducer drug discontinuation were fitted to a one-phase decay model and the half-life was calculated as t1/2≈ 3.2 h. (**f, g**) Inducible CAR T cells bearing

one to five binding sites for the synthetic transcription factor, direct CAR T cells and untransduced cells were stimulated with TransAct and induced with 100 nM 4-OHT. Cell surface expression of anti-CD20 CAR was determined after 48 h by flow cytometry using anti-CD20 CAR detection reagent-PE. Data for frequency (**f**) and MFI (**g**) of anti-CD20 CAR T cells are normalized to the expression of the transduction marker ΔLNGFR. Graphs show data from $n = 3$ (c, d, e); $n = 2$ (**f, g**) different donors. Data shown are mean values ± s.d. with $^{*}p < 0.05$, $^{**}p < 0.01$, $^{***}p < 0.001$, $^{****}p < 0.0001$ by two-way analysis of variance (ANOVA).

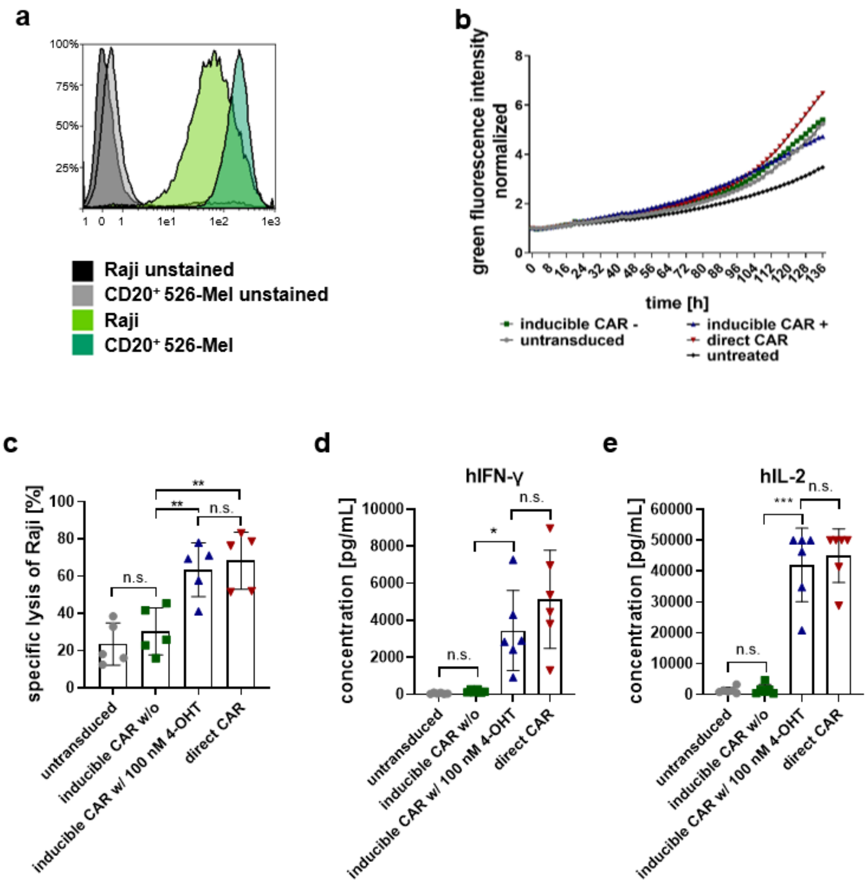

Figure S2. Cytotoxic activity of inducible anti-CD20 CAR T cells against Raji and antigen negative 526-Mel cells in vitro. (**a**) Flow histogram depicting the cell surface staining of the tumor cell lines GFP+ Raji and GFP+ CD20+ 526-Mel for the target antigen CD20 using anti-CD20-PE. (**b**) Inducible CAR T cells were co-cultured with antigen negative GFP+ 526-Mel at an E:T ratio of 1:1 in the absence (-) or presence of 100 nM 4-OHT (+) added at the start of the assay. Growth of GFP+ CD20+ 526-Mel was monitored in 2 h intervals using a live-cell imaging device. (**c**, **d, e**) Inducible CAR T cells comprising five binding sites for the synthetic transcription factor were co-cultured with GFP+ Raji at an E:T ratio of 1:1 in the presence of 100 nM 4-OHT added at the start of the assay. (**c**) Specific lysis of tumor cells was measured 40 h after co-culture setup by the quantification of living GFP+ Raji via MACS Quant Analyzer. (**d**, **e**) The concentration of human IFN-γ (**d**) and IL-2 (**e**) was analyzed in the supernatant of 40 h co-cultures by MACSPlex Cytokine 12 Kit. Graphs show data from $n = 1$ (**b**), $n = 5$ (**c**), $n = 6$ (**d**, **e**), different donors. Data shown are mean values ± s.d. with $^{*}p < 0.05$, $^{**}p < 0.01$, $^{***}p < 0.001$, $^{****}p < 0.0001$ by one-way analysis of variance (ANOVA).

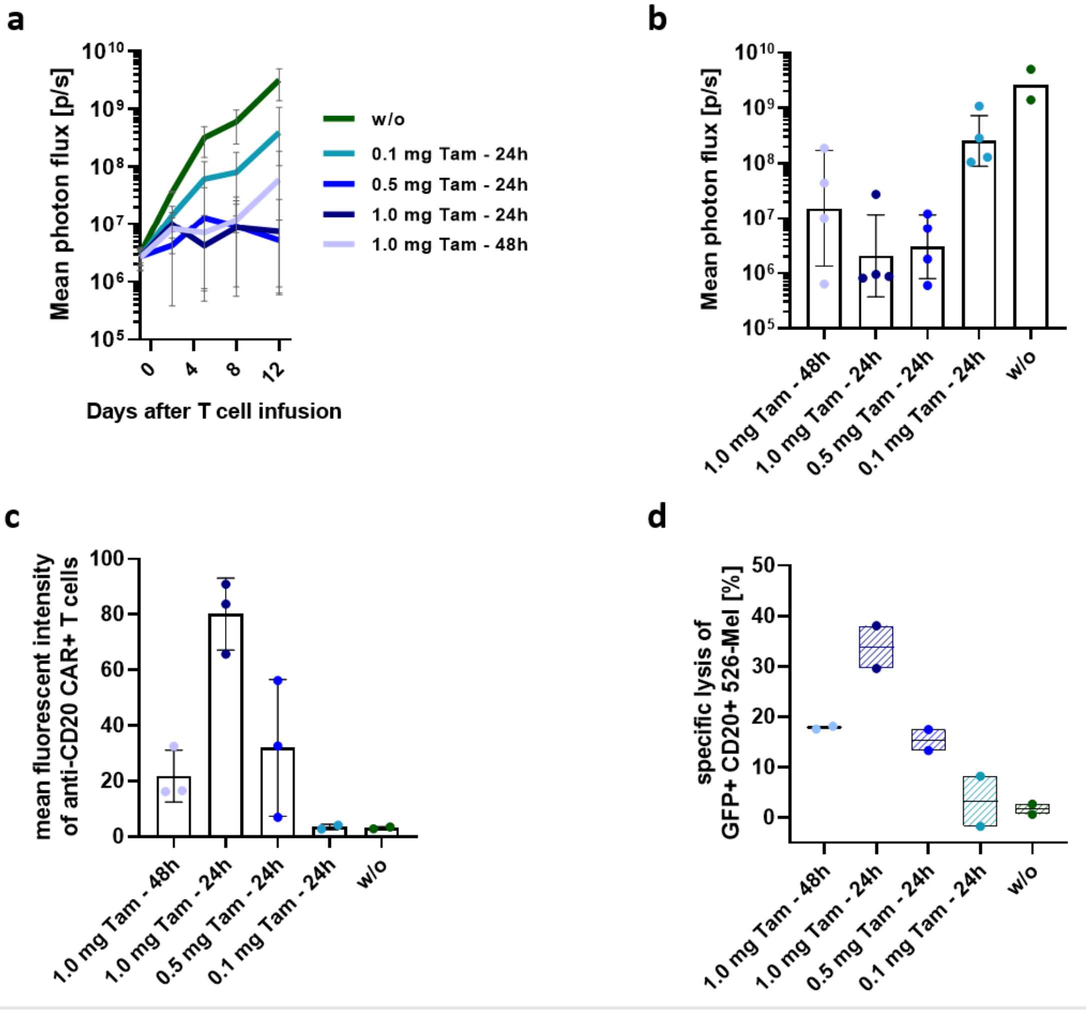

Figure S3. Modulation of inducible anti-CD20 CAR T cells in vivo by inducer dose and injection interval**.** NSG mice were inoculated with 4×10^5 Raji^{ffLuc} cells via tail-vein injection on day -5. On day 0, following randomization, mice were treated with 3×10^6 inducible anti-CD20 CAR T cells and a total of 1.1×10^7 total T cells. Individual groups received 1, 0.5 or 0.1 mg tamoxifen either at a 24 h or 48 h administration interval by i.p. injections starting on day -1. (**a**) Tumor burden expressed in mean photon flux [photons/sec] was determined by in vivo BLI for individual inducible CAR groups on day -1 (randomization) and days 2, 5, 8, and 12 after T cell injection **(b)** Photon flux [photons/sec] of individual mice plotted for day 12 after T cell injection. (**c**) For ex vivo analysis, cells were isolated from the bone marrow of femur and tibia either on day 12 (0.1 mg - 24 h, w/o) or on day 20 (1 mg - 48 h, 1 mg – 24 h, 0.5 mg – 24 h) after T cell infusion. Isolated cells were stained for anti-CD20 CAR expression using the anti-CD20 CAR detection reagent and analyzed by flow cytometry. MFI of anti-CD20 CAR$^+$ T cells is plotted for individual mice. (**d**) Inducible CAR T cells were co-cultured with GFP$^+$ CD20$^+$ 526-Mel in the presence of 10 µL plasma from mice treated with tamoxifen at indicated doses and injection intervals. Specific lysis of GFP$^+$ CD20$^+$ 526-Mel was measured 40 h after co-culture setup.

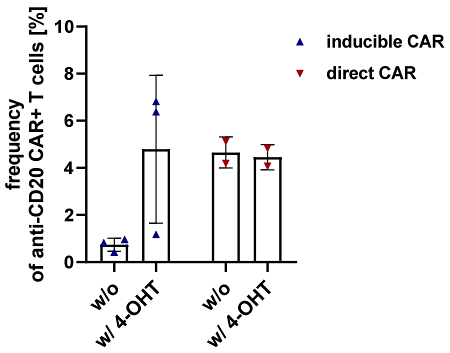

Figure S4. Induction of anti-CD20 CAR expression in ex vivo cultivated T cells Ex vivo cultivated T cells isolated via CD4⁺/CD8⁺ enrichment from the spleen of individual mice were induced with 100 nM 4-OHT for 46 h and analyzed for anti-CD20 CAR expression by flow cytometry. Data represent the frequency of anti-CD20 CAR expressing cells.

Table S1. Comparison of transduction frequencies of inducible and conventional (direct) anti-CD20 CAR T cells.

	**inducible anti-CD20 CAR**	**direct anti-CD20 CAR**
frequency of LNGFR$^+$ T cells post enrichment [%]	30.1 ± 10.9 (range 18.1 – 52.8, n=10)	94.4 ± 1.9 (range 90.9 – 97.0, n=10)

Frequencies of LNGFR$^+$ T cells post enrichment include data from n = 10 different donors.
